# Supplementary material for: Electron-Induced Decomposition of Uracil-5-yl O-(N,N-dimethylsulfamate): Role of Methylation in Molecular Stability
Source: Int J Mol Sci. 2021 Feb 26;22(5):2344. doi: 10.3390/ijms22052344 (PMC7956691; doi:10.3390/ijms22052344)
Supplement: Supplementary file 1 [file ijms-22-02344-s001.pdf]

# Supplementary Materials

**Table of Content**

$^{1}H$ NMR spectrum of DMSU S2

MS spectrum of DMSU S2

MS/MS spectrum of DMSU S3

UV-Vis absorption spectrum of DMSU S3

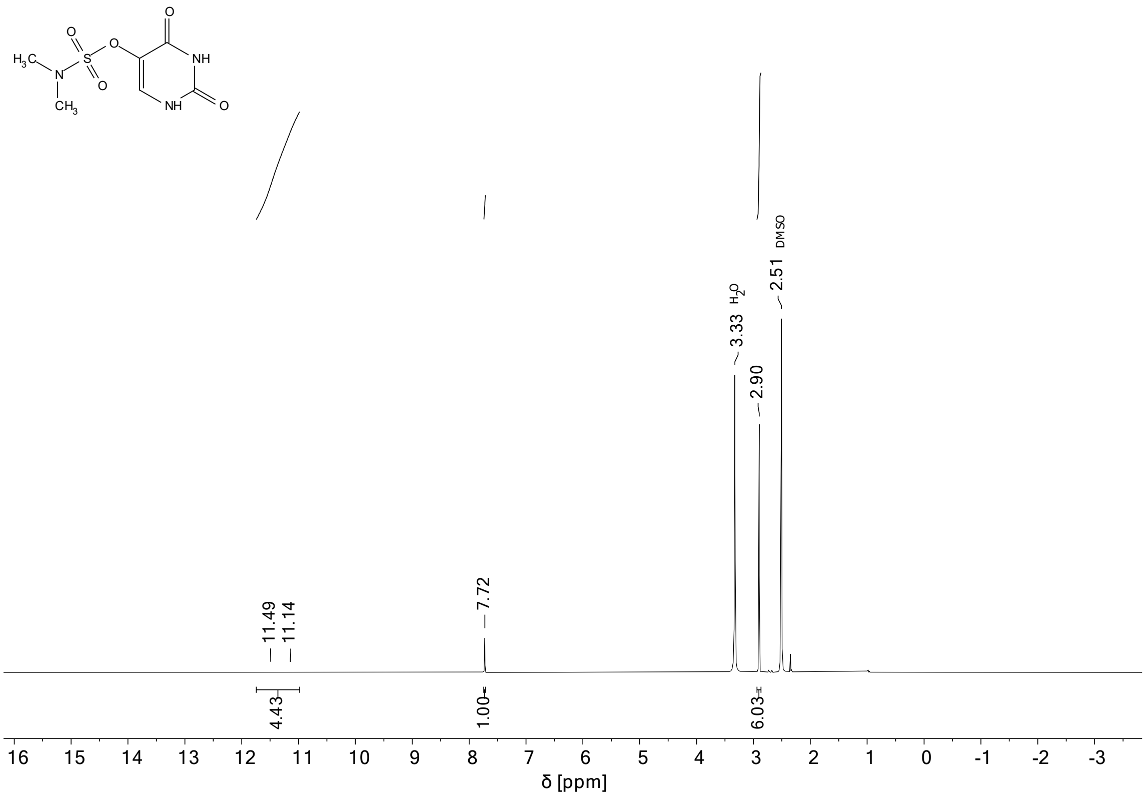



Figure S1. The ¹H NMR spectrum of DMSU.

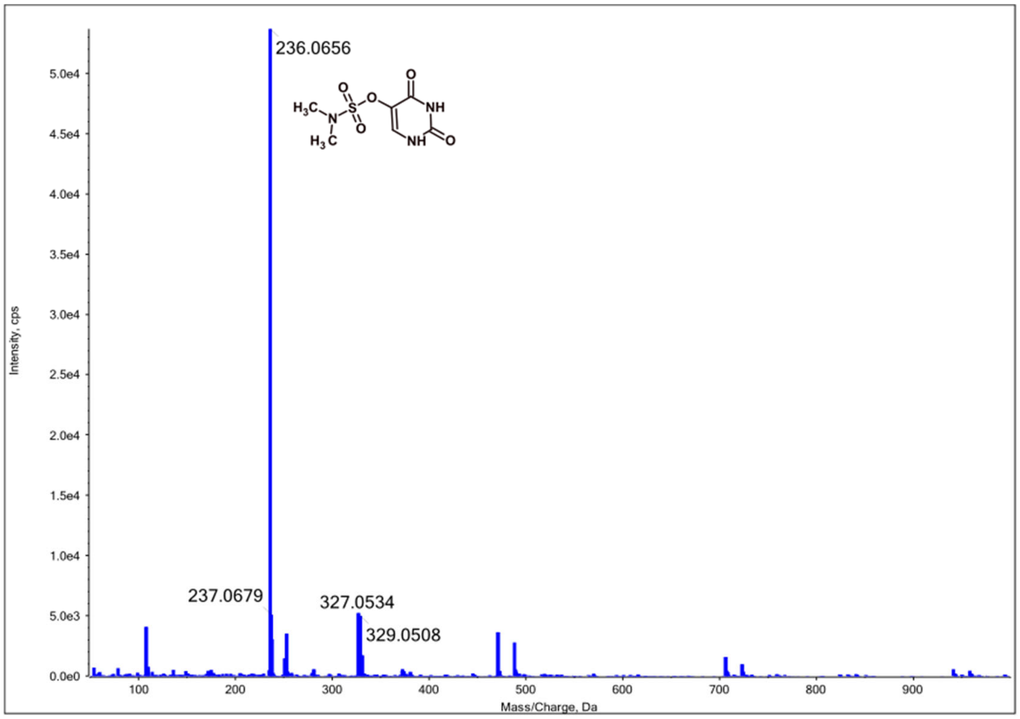



Figure S2. The MS spectrum (in positive ionization mode) of DMSU.

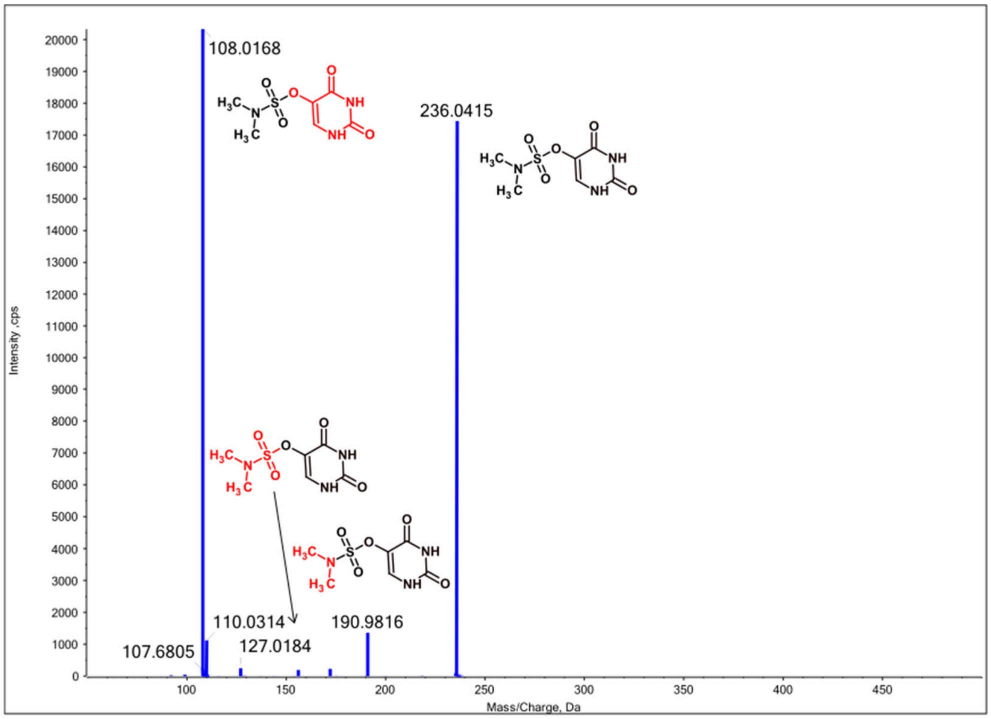


Figure S3. The MS/MS spectrum (in positive ionization mode) of DMSU and ion identities. The red color indicates the molecular fragments released during fragmentation. The signals observed in the MS/MS spectrum correspond to the molecular fragments shown in black.

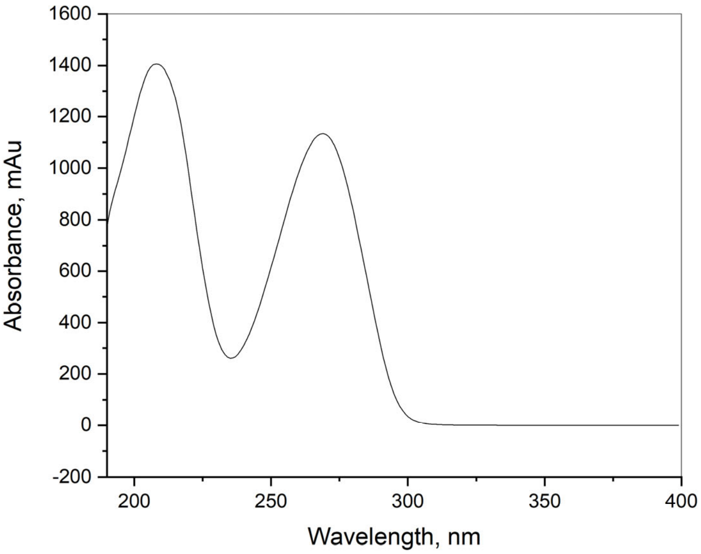


Figure S4. The UV-Vis absorption spectrum of DMSU.
